# Efficacy and Safety of Radioligand Therapy with Actinium-225 DOTATATE in Patients with Advanced, Metastatic or Inoperable Neuroendocrine Neoplasms: A Systematic Review and Meta-Analysis

**DOI:** 10.3390/medicina61081341

**Published:** 2025-07-24

**Authors:** Alessio Rizzo, Alessio Imperiale, Salvatore Annunziata, Roberto C. Delgado Bolton, Domenico Albano, Francesco Fiz, Arnoldo Piccardo, Marco Cuzzocrea, Gaetano Paone, Giorgio Treglia

**Affiliations:** 1 Department of Nuclear Medicine, Candiolo Cancer Institute, FPO-IRCCS, 10060 Turin, Italy; alessio.rizzo@ircc.it; 2 Nuclear Medicine, University Hospitals of Strasbourg, University of Strasbourg, 67200 Strasbourg, France; a.imperiale@icans.eu; 3 Nuclear Medicine and Molecular Imaging, ICANS, 67200 Strasbourg, France; 4 Nuclear Medicine Unit, Fondazione Policlinico Universitario A. Gemelli IRCCS, 00168 Rome, Italy; salvatore.annunziata@policlinicogemelli.it; 5 Department of Diagnostic Imaging (Radiology) and Nuclear Medicine, University Hospital San Pedro and Centre for Biomedical Research of La Rioja (CIBIR), 26006 Logroño, Spain; rbiolton@gmail.com; 6 Servicio Cántabro de Salud, 39011 Santander, Spain; 7 Department of Nuclear Medicine, ASST Spedali Civili di Brescia and University of Brescia, 25123 Brescia, Italy; domenico.albano@unibs.it; 8 Department of Nuclear Medicine, Ente Ospedaliero Ospedali Galliera, 16128 Genoa, Italy; francesco.fiz@galliera.it (F.F.); arnoldo.piccardo@galliera.it (A.P.); 9 Division of Nuclear Medicine, Imaging Institute of Southern Switzerland, Ente Ospedaliero Cantonale, 6500 Bellinzona, Switzerland; marco.cuzzocrea@eoc.ch (M.C.); gaetano.paone@eoc.ch (G.P.); 10 Faculty of Biomedical Sciences, Università della Svizzera italiana, 6900 Lugano, Switzerland; 11 Faculty of Biology and Medicine, University of Lausanne, 1005 Lausanne, Switzerland

**Keywords:** neuroendocrine, radioligand therapy, Actinium-225, DOTATATE, alpha-emitting, somatostatin, nuclear medicine, meta-analysis, safety, efficacy

## Abstract

*Background and Objectives*: Peptide receptor radionuclide therapy (PRRT) using radiopharmaceuticals labelled with Lutetium-177 is currently a therapeutic option for patients with advanced neuroendocrine neoplasms overexpressing somatostatin receptors (SSTRs). One promising option that has gained interest for PRRT is using alpha-emitting radioisotopes such as Actinium-225. The aim of this study was to perform a systematic review and meta-analysis on the efficacy and safety of radioligand therapy with Actinium-225 DOTATATE in advanced, metastatic or inoperable neuroendocrine neoplasms. *Materials and Methods:* A comprehensive literature search of studies on radioligand therapy with Actinium-225 DOTATATE in neuroendocrine neoplasms was carried out. Three different bibliographic databases (Cochrane Library, Embase, and PubMed/MEDLINE) were screened up to May 2025. Eligible articles were selected, relevant data were extracted, and the main findings on efficacy and safety are summarized through a systematic review. Furthermore, proportional meta-analyses on the disease response rate and disease control rate were performed. *Results*: Five studies (153 patients) published from 2020 were included in the systematic review. The pooled disease response rate and disease control rate of radioligand therapy using Actinium-225 DOTATATE were 51.6% and 88%, respectively. This treatment was well-tolerated in most patients with advanced, metastatic or inoperable neuroendocrine neoplasms. *Conclusions*: Radioligand therapy with Actinium-225 DOTATATE in advanced, metastatic or inoperable neuroendocrine neoplasms is effective with an acceptable toxicity profile and potential advantages compared with SSTR-ligands labelled with Lutetium-177. Currently, the number of published studies on this treatment is still limited, and results from multicenter randomized controlled trials are needed to translate this therapeutic option into clinical practice.

## 1. Introduction

Neuroendocrine neoplasms are heterogeneous tumors originating from neuroendocrine cells and can occur anywhere in the body. The incidence of these neoplasms is increasing worldwide. Neuroendocrine neoplasms comprise a broad family of tumors based on their origin, location, and tumor grade (related to the proliferation assessed by mitotic rate and Ki-67 labeling index). Some neuroendocrine neoplasms are characterized by hormonal hypersecretion causing functional abnormalities and related symptoms, whereas others are non-functional tumors. Overall, diagnosis and treatment of neuroendocrine neoplasms depend on tumor type, location, grade, hormonal hypersecretion, and metastatic spread [1]. As heterogeneous diseases, neuroendocrine neoplasms require multidisciplinary treatment, including medical therapy, surgery, chemotherapy, and radiation therapy including radioligand therapy [1].

Most neuroendocrine neoplasms are characterized by overexpression of somatostatin receptors (SSTRs), which provides the rationale for using radiolabeled SSTR ligands for diagnosis and therapy of these tumors through nuclear medicine procedures. Theranostic approaches integrating SSTR-targeted imaging and radioligand therapy have been evaluated in patients with neuroendocrine neoplasm for personalized cancer therapy [2,3,4,5,6,7,8,9,10,11].

Peptide receptor radionuclide therapy (PRRT) with radiolabeled SSTR ligands is currently indicated as second- or third-line therapy for metastatic or unresectable, progressive, SSTR-positive neuroendocrine neoplasms [11]. Currently, the most frequent radiopharmaceuticals used for PRRT in neuroendocrine neoplasms are SSTR ligands labeled with Lutetium-177 (i.e., Lutetium-177 DOTATATE), which enables high-energy short-range deposits in tissues, causing selective irradiation of the targeted tumor lesions [12]. It has been clearly demonstrated that PRRT with Lutetium-177 DOTATATE prolongs progression-free and overall survival in patients with advanced, metastatic, or inoperable neuroendocrine neoplasms, reduces or stabilizes the tumor burden, and improves tumor symptoms and quality of life. Adverse events associated with this treatment are mostly mild and transient [11].

However, according to evidence-based data, only 20–30% of patients with advanced, metastatic or inoperable neuroendocrine neoplasms achieved a disease response (including partial or complete response) to PRRT and most patients relapsed within 2–3 years of treatment [13,14,15]. Therefore, alternative strategies for PRRT in neuroendocrine neoplasms have been evaluated.

One promising option that has gained interest for PRRT is using alpha-emitting radioisotopes such as Actinium-225, characterized by high linear energy transfer compared with beta-emitting radioisotopes such as Lutetium-177. Due to theoretical physical advantages related to alpha-emitting isotopes, PRRT with SSTR ligands labelled with Actinium-225 (i.e., Actinium-225 DOTATATE) is expected to further improve the efficacy and safety of PRRT in advanced, metastatic, or inoperable neuroendocrine tumors compared with SSTR ligands labelled with lutetium-177 [12]. Indeed, Actinium-225 has a long half-life of about ten days and undergoes multiple decay steps before reaching a stable form (Thallium-205); in each of these steps, a high-energy alpha particle or a beta electron is emitted [16]. Alpha particles are helium cores that, due to their size, have a greater chance of causing double-strand DNA breaks in the target cell nucleus, which is more challenging to recover from, especially at higher doses [17].

The aim of this evidence-based article is to summarize the literature data about the efficacy and safety of radioligand therapy with Actinium-225 DOTATATE in patients with advanced, metastatic or inoperable neuroendocrine neoplasms through a systematic review and a meta-analysis.

## 2. Materials and Methods

### 2.1. Review Question

This systematic review and meta-analysis were conducted and written according to a predefined protocol (but without registration in a public database) following the Preferred Reporting Items for Systematic Reviews and Meta-analyses (PRISMA) guidelines [18]. The full PRISMA checklist is available in the Supplementary Material.

The following review question was formulated by the authors based on the PICO (patients/intervention/comparison/outcome) framework: “What are the efficacy and safety of radioligand therapy with Actinium-225 DOTATATE in patients with advanced, metastatic or inoperable neuroendocrine neoplasms?”.

Within the design of the review question, the presence of a comparator was not deemed as exclusion criteria, as studies aiming to evaluate the overall efficacy and safety of a specific intervention might not compare their results with those of an alternative treatment.

### 2.2. Search Strategy

Three different bibliographic databases (Cochrane Library, Embase and PubMed/MEDLINE) were screened by two review authors (A.R. and G.T.) independently (last date of screening was 25 May 2025).

The review authors created a search string for each database based on the combination of the following key words: (A) “neuroendocrine” OR “NET” OR “NEN” AND (B) “Actinium” OR “Actinium-225” OR “Ac-225” OR “Ac225” OR “225Ac” OR “225Ac-DOTA*” OR “Ac-225-DOTA*” OR “Ac225-DOTA*” OR “Ac-DOTA*” OR “RYZ101”. For the literature search, no language or publication date restrictions were applied.

Reference lists of potentially eligible studies were also screened to identify additional studies enhancing the sensitivity of the literature search.

### 2.3. Selection of Studies

The study selection was independently carried out by two authors (A.R., and G.T.), applying the following predefined inclusion and exclusion criteria related to the review questions:

- Inclusion criteria for the systematic review were studies or subsets of studies investigating the efficacy and/or safety of radioligand therapy with Actinium-225 DOTATATE in patients with advanced, metastatic or inoperable neuroendocrine neoplasms.
- Exclusion criteria for the systematic review were articles outside the field of interest, preclinical studies, editorials, letters, reviews, comments, conference proceedings, and case reports related to the selected intervention.

In the context of our review, which analyzes a novel topic in which the available literature generally consists of early-phase or proof-of-concept studies, the objective was to synthesize all relevant evidence regarding Actinium-225 DOTATATE, regardless of whether a direct comparison was included. Therefore, the authors applied broad inclusion criteria and included studies of various designs (prospective, retrospective, cohort-based) that included administering Actinium-225 DOTATATE for the treatment of advanced, metastatic or inoperable neuroendocrine neoplasms. Given the constrained nature of the current evidence, this approach was intended to maximize sensitivity rather than specificity.

The titles and abstracts of the records retrieved were initially screened by two authors. After excluding ineligible records, the full texts of potentially eligible articles were downloaded and reviewed. Lastly, studies were included in the review following a consensus meeting among all the co-authors.

Articles included in the systematic review were eligible for the meta-analysis whether sufficient data to calculate the predefined outcomes were available.

### 2.4. Data Extraction and Quality Assessment

Data extraction and quality assessment were carried out by two review authors (A.R. and G.T.) independently. Data extracted from the selected studies included fundamental study details (authors, publication year, country, study design), patient characteristics (number of cases, mean or median age, sex ratio, type of tumor, and tumor grade), technical aspects of the intervention, and outcome data regarding the efficacy and safety of the intervention. NIH quality assessment tools were used for the risk of bias/quality assessment of the selected studies [19].

### 2.5. Statistical Analysis

Disease control rate (defined as the percentage of patients with advanced, metastatic or inoperable neuroendocrine neoplasms who have achieved complete response, partial response and stable disease to the intervention) and disease response rate (defined as the percentage of patients with advanced, metastatic or inoperable neuroendocrine neoplasms who have achieved complete or partial response to the intervention) were calculated for each selected study. The pooled outcome measures were obtained through a proportional patient-based meta-analysis using a random-effects model. This statistical model considers the variability among studies [20]. Subgroup analyses were performed if sufficient data were available from the included studies and in case of significant statistical heterogeneity. Pooled data were presented with a summary effect measure and 95% confidence interval (95%CI) values. Statistical heterogeneity was estimated through the I^2^ statistic [20]. OpenMeta[Analyst] (version 1.0 for Windows) was used as statistical software for the meta-analysis.

## 3. Results

### 3.1. Literature Search

**Figure 1:**
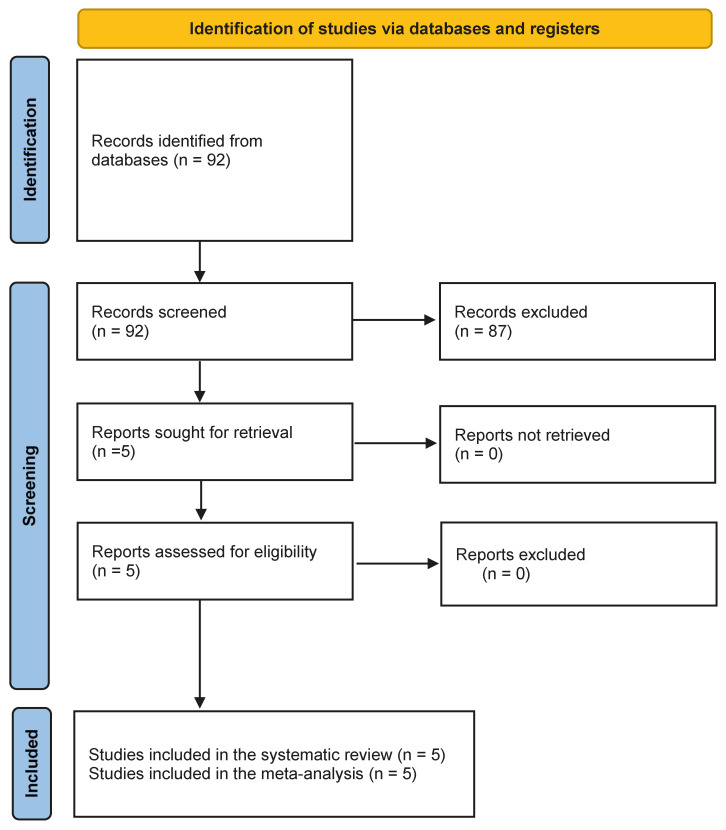
PRISMA flow chart about the selection of studies.

A total of 92 records were identified and screened using the selected bibliographic databases; 87 reports were excluded according to the exclusion criteria previously mentioned and five studies (153 patients) investigating the efficacy and safety of radioligand therapy with Actinium-225 DOTATATE in patients with advanced, metastatic, or inoperable neuroendocrine neoplasms were finally included in the systematic review and meta-analysis [21,22,23,24,25], without additional studies identified after screening the reference lists of the retrieved articles. The main characteristics of the included studies and related results are illustrated in Table 1 and Table 2. The PRISMA flow chart is reported in Figure 1.

### 3.2. Quality Assessment

All the five selected studies showed an overall moderate quality according to the NIH quality assessment tool, and 80% of the included articles were prospective studies [21,22,23,24,25].

### 3.3. Qualitative Synthesis

Studies on Actinium-225 DOTATATE in advanced, metastatic or inoperable neuroendocrine neoplasms were published since the year 2020 by researchers from India (*n =* 3), China (*n =* 1), and Turkey (*n =* 1) [21,22,23,24,25].

A total of 153 patients were enrolled in the included studies (range: 9*–*91). Median/mean age ranged from 41 to 59 years and the sex ratio varied among the included studies [21,22,23,24,25].

Various advanced, metastatic or inoperable neuroendocrine neoplasms were treated with Actinium-225 DOTATATE, including cases with different origins (gastrointestinal, pancreatic, neural crest, lung, thyroid and unknown origin) and tumor grade. The included patients had inoperable neuroendocrine neoplasms, postoperative tumor recurrence, or distant metastases. Both treatment-naive patients or those resistant to conventional therapies were included. Patients previously treated or not treated with Lutetium-177 DOTATATE were enrolled [21,22,23,24,25].

The following patients were not suitable for radioligand therapy with Actinium-255 DOTATATE: those with low baseline hemoglobin level or thrombocytopenia or leukopenia, those with renal insufficiency, hepatic insufficiency, life expectancy of less than 6 months, or low performance status [21,22,23,24,25].

Before performing radioligand therapy with Actinium-225 DOTATATE, the increased expression of SSTR type 2 receptors by tumor lesions was demonstrated by SSTR PET/CT. Actinium-225 DOTATATE treatment was repeated after an 8-week interval. In the included studies, the dose per cycle was 100*–*120 kBq/kg, the median number of cycles ranged from 1 to 4, and the median cumulative activity ranged from 8.2 to 42.4 MBq. The treatment was discontinued if the patients demonstrated disease progression during treatment. Amino acids were infused before administration of Actinium-225 DOTATATE, to protect the kidneys. Antiemetics and corticosteroids were administered before the amino acid infusion to prevent nausea and vomiting, and the drugs were repeated as necessary [21,22,23,24,25].

A complete blood cell count, kidney function test, and liver function test were performed before and after each cycle of Actinium-225 DOTATATE for toxicity assessment. Morphological and functional imaging was performed after treatment to assess the efficacy of treatment and restage the disease. Treatment response assessment was performed according to the Response Evaluation Criteria in Solid Tumors (RECIST 1.1) criteria or Positron Emission Tomography Response Criteria in Solid Tumors (PERCIST 1.0) criteria. The median follow-up time in the included studies ranged from 8 to 24 months [21,22,23,24,25].

Regarding the main outcomes of treatment response, the disease control rate ranged from 80% to 100% in the included studies, whereas the disease response rate ranged from 40% to 62.5% [21,22,23,24,25]. The median overall survival and progression-free survival were not reached or were not calculated in most of the studies; a long-term outcome study reported the 24-month overall survival rate and the probability of progression-free survival to be 70.8% and 67.5%, respectively [22].

In terms of its safety, radioligand therapy with Actinium-225 DOTATATE was well tolerated. Regarding serious adverse events, only one case of grade III thrombocytopenia was reported in one study; therefore, the treatment was well-tolerated. Renal function was well preserved across all studies; specifically, Yang and colleagues reported a single case of grade II renal toxicity [21], and Demirci and colleagues reported one case of renal toxicity in a patient who had been heavily pre-treated with Lutetium-177-DOTATATE [23]. The most common adverse events recorded in the included studies were loss of appetite, nausea, and vomiting. Overall, most symptoms improved after Actinium-225 DOTATATE treatment [21,22,23,24,25].

Clinical response in metastatic or advanced NEN was assessed in the studies by Yang et al. and Ballal et al. using the European Organization for Research and Treatment of Cancer Core Quality of Life questionnaire (EORTC-QLQ) [21,25]. Most symptoms improved after Actinium-225 DOTATATE treatment [21,25], with only diarrhea showing no improvement [21]. In addition to morphologic responses, improvements in overall patient quality of life after Actinium-225 DOTATATE treatment was observed also in the study by Ballal et al., with the median Karnofsky performance status (KPS) increasing from 60 before treatment (the patient requiring medical care and much assistance with self-care) to 70 post-treatment (the patient being able to care for themselves but being unable to do their usual activities or active work) [22]. A notable improvement was also observed after Actinium-225 DOTATATE treatment in the study by Yadav et al. that focused on advanced paragangliomas [24].

### 3.4. Quantitative Synthesis (Meta-Analysis)

**Figure 2:**
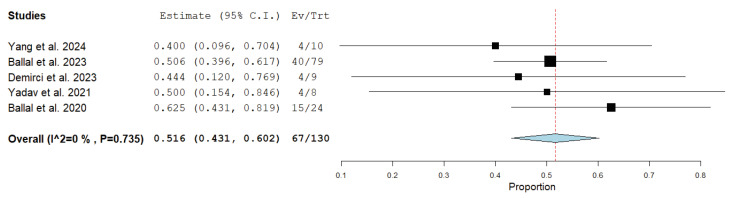
Forest plot of the meta-analysis on the disease response rate using Actinium-255 DOTATATE in advanced, metastatic or inoperable neuroendocrine neoplasms. The outcome measure is reported as a square for each included study. The horizontal lines represent the 95% confidence interval values. The size of the square is related to the weight of each study. The diamond represents the pooled outcome measure and the long axis of the diamond represents the pooled 95% confidence interval values [21,22,23,24,25].

**Figure 3:**
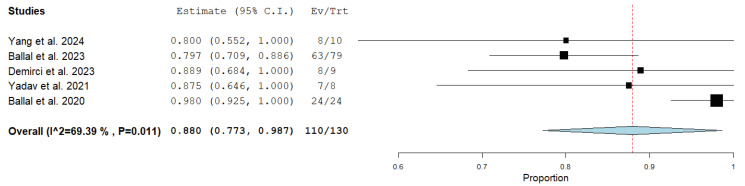
Forest plot of the meta-analysis on the disease control rate using Actinium-255 DOTATATE in advanced, metastatic or inoperable neuroendocrine neoplasms. The outcome measure is reported as a square for each included study. The horizontal lines represent the 95% confidence interval values. The size of the square is related to the weight of each study. The diamond represents the pooled outcome measure and the long axis of the diamond represents the pooled 95% confidence interval values [21,22,23,24,25].

Results of the meta-analysis about the treatment outcome of Actinium-225 DOTATATE in patients with advanced, metastatic or inoperable neuroendocrine neoplasms are reported in Figure 2 and Figure 3. Due to the available information, only 130 out of 153 patients enrolled in the included studies were included in the meta-analysis. Radioligand therapy with Actinium-225 DOTATATE in these patients demonstrated a pooled disease control rate of 88% (95%CI: 77.3–98.7%) and a pooled disease response rate of 51.6% (95%CI: 43.1–60.2%). Moderate statistical heterogeneity was found only according to the meta-analysis on the disease control rate (I^2^ = 69%) and not the meta-analysis on the disease response rate (I^2^ = 0%). Due to limited available data, subgroup analyses were not performed.

## 4. Discussion

Radioligand therapy, particularly PRRT, has garnered significant interest following the advent of novel theranostic compounds, which have shown encouraging results across various cancer types, including neuroendocrine neoplasms [12,26].

While the advent and regulatory approval of PRRT with Lutetium-177 DOTATATE has offered a valuable therapeutic option for patients with neuroendocrine neoplasms, significant opportunities still exist to unlock the full therapeutic potential of PRRT in these tumors [27].

Overall, several literature data demonstrate that alpha-emitting radiopharmaceuticals such as Actinium-225 DOTATATE offer a potential advantage over beta-emitting radiopharmaceuticals such as Lutetium-177 DOTATATE, due to higher cytotoxicity related to their greater linear energy transfer and the ability to induce complex DNA damage (including double-stranded breaks) and targeted cell damage [27,28,29,30,31,32,33,34,35,36,37,38,39].

Furthermore, alpha-emitting radiopharmaceuticals have the potential to reduce off-target toxicity while maintaining high tumor-killing efficacy, due to the shorter path length of alpha particles compared with beta particles [27,28,29,30,31,32,33,34,35,36,37,38,39].

Existing pre-clinical and clinical data seem to suggest promising efficacy and safety of Actinium-225 DOTATATE as a novel alpha-emitting SSTR- targeting radiopharmaceutical [26]. Due to the increasing interest in this topic, we carried out this systematic review and meta-analysis to provide updated information, higher statistical power, and more precise estimates of the safety and efficacy of this therapeutic option compared with single studies (usually including small patient populations). We also excluded case reports from our analysis due to their intrinsic biases.

We found that, to date, five original studies, mostly prospective, have reported outcome measures on safety and efficacy of Actinium-225 DOTATATE in advanced, metastatic or inoperable neuroendocrine neoplasms [21,22,23,24,25].

Pooled data provided by our analysis demonstrate a significant efficacy of this treatment with a pooled disease response rate and disease control rate of 52% and 88%, respectively. Notably, these values are significantly higher than those obtained using Lutetium-177 DOTATATE in the same tumors [13,14,15]. Interestingly, Actinium-225 DOTATATE has also been effective in patients with neuroendocrine neoplasms previously treated with SSTR ligands labelled with Lutetium-177 [19,20,21,22,23], thus suggesting that this treatment could be an option for patients with stable disease or disease progression after Lutetium-177 DOTATATE [20]. Other factors, including demographic characteristics, did not appear to influence the outcome of the treatment with the alpha emitter.

Regarding safety, serious adverse events of Actinium-225 DOTATATE in advanced, metastatic or inoperable neuroendocrine neoplasms are rare [21,22,23,24,25], thus confirming its excellent safety profile compared with Lutetium-117 DOTATATE [27]. Also, quality of life significantly improved after treatment with Actinium-225 DOTATATE [21,22,23,24,25].

Although interesting, the findings of our systematic review and meta-analysis need to be confirmed by further well-designed studies. Currently, PRRT with Actinium-225 DOTATATE still remains an investigational treatment for patients with advanced, metastatic or inoperable neuroendocrine neoplasms.

The ongoing ACTION-1 trial is comparing the efficacy and safety of Actinium-225 DOTATATE against standard care therapies in advanced, metastatic or inoperable neuroendocrine neoplasms after disease progression following prior radioligand therapy with SSTR ligands labelled with Lutetium-177 [27].

Additionally, although one study reported promising long-term results of Actinium-225 DOTATATE in advanced, metastatic or inoperable neuroendocrine neoplasms with transient and acceptable adverse effects [22], a meta-analysis on overall survival and progression-free survival as outcome measures was not feasible and more data on the long-term efficacy and safety of Actinium-225 DOTATATE are warranted.

Furthermore, other alpha-emitting radiopharmaceuticals beyond Actinium-225 DOTATATE are currently being investigated in advanced, metastatic or inoperable neuroendocrine neoplasms, with promising results [27,40]. Specifically, radiopharmaceuticals based on Astatine-211 could be attractive due to the low half-life of this radioisotope, which would allow easier adjustment of the dose to the target and organs at risk [28]. Lead-212 is another promising candidate due to the particularly high energy of its alpha particle emission [41]. Other future developments include dosimetry studies which could help optimize treatment delivery and minimize toxicity, and combinations of PRRT with alpha-emitting radiopharmaceuticals and radiation sensitizers, DNA damage repair inhibitors, or immunotherapy to increase treatment efficacy and prolong treatment responses [25]. Also, the clinical implementation of new imaging biomarkers, especially based on multitracer PET/CT imaging, may improve patient management [42].

Beyond efficacy and safety, cost-effectiveness of treatment is another outcome that should be evaluated before the clinical implementation of novel therapies. While cost-effectiveness analyses on Lutetium-177 DOTATATE in neuroendocrine neoplasms in specific scenarios are already available [43], no cost-effectiveness data have been published on Actinium-225 DOTATATE.

The main limitations of our systematic review and meta-analysis include the low number of included studies and the heterogeneity of patients, disease characteristics, and treatment. Consistent with the constrained number of the included studies and the subsequent small sample analyzed, selection bias cannot be excluded. An additional methodological limitation of this study was the absence of a comparative analysis between Actinium-225 DOTATATE and alternative treatments. The present meta-analysis was conducted as a single-arm synthesis, aiming to summarize the efficacy and safety outcomes of Actinium-225 DOTATATE independently, due to the limited number of available studies and the heterogeneity in trial designs. While some statistically significant findings were observed within the Actinium-225 group, these cannot be interpreted as evidence of superiority or inferiority in comparison to other therapeutic options (such as Lutetium-177 DOTATATE) as no formal intergroup comparison was performed. Consequently, future studies including direct or indirect comparisons will be essential to more accurately position Actinium-225 DOTATATE within the therapeutic landscape for neuroendocrine neoplasms. However, we provide useful pooled estimates on the efficacy of this novel treatment, and the clinical and methodological heterogeneity observed in the studies included in our analysis did not lead to significant statistical heterogeneity.

## 5. Conclusions

Overall, our systematic review and meta-analysis demonstrated that radioligand therapy with Actinium-225 DOTATATE in advanced, metastatic or inoperable neuroendocrine neoplasms is effective with an acceptable toxicity profile and potential advantages compared with SSTR ligands labelled with Lutetium-177. This treatment could be a suitable therapeutic option for patients refractory to SSTR ligands labelled with Lutetium-177. Currently, the number of published studies is still limited, and results from multicenter randomized controlled trials and cost-effectiveness studies are needed for translating this therapeutic option for neuroendocrine neoplasms into clinical practice.

## Figures and Tables

**Table 1:** Basic study and patient characteristics.

Authors	Year	Country	Study Design	No. of Cases	Mean or Median Age	Sex Ratio	Types of Tumor	Tumor Grade (Based on Ki-67%)
Yang et al. [21]	2024	China	Prospective	10	47.5 y	3 F/7 M	Various metastatic NENs	I = 10%, II = 80%; NA = 10%
Ballal et al. [22]	2023	India	Prospective	91	54 y	37 F/54 M	Metastatic GEP-NENs	I = 36%, II = 53%; III = 8%; NA = 3%
Demirci et al. [23]	2023	Turkey	Retrospective	11	59 y	3 F/8 M	Various metastatic NENs	I = 20%, II = 70%; NA = 10%
Yadav et al. [24]	2022	India	Prospective	9	41 y	3 F/6 M	Advanced PGLs	I = 33%, II = 33%; III = 11%; NA = 22%
Ballal et al. [25]	2020	India	Prospective	32	52 y	17 F/15 M	Metastatic GEP-NENs	I = 34%, II = 50%; III = 9%; NA = 6%

Legend: y = years; F = female; M = male; GEP = gastroenteropancreatic; NA = not available; NENs = neuroendocrine neoplasms; PGLs = paragangliomas.

**Table 2:** Characteristics of intervention and related outcomes.

Authors	Radiopharmaceutical	Median Number of Cycles (Range)	Dose per Cycle and Interval	Median/Mean Cumulative Activity (Range)	Treatment Response Criteria	Median Follow-Up Time (Range)	DCR	DRR	Serious Adverse Events
Yang et al. [21]	^225^Ac-DOTATATE	3 (2–6)	100 kBq/kg; 8-week interval	22.9 MBq (14.8–44.4)	PERCIST 1.0	14 months (7–22)	8/10 (80%)	4/10 (40%)	None
Ballal et al. [22]	^225^Ac-DOTATATE	4 (1–10)	100–120 kBq/kg; 8-week interval	35.5 MBq (21.6–59.5)	RECIST 1.1	24 months (5–41)	63/79 (80%)	40/79 (51%)	Grade III thrombocytopenia (1/91)
Demirci et al. [23]	^225^Ac-DOTATATE	1 (1–3)	100–120 kBq/kg; interval NR	8.2 MBq (7.5–10)	RECIST 1.1	NR	8/9 (89%)	4/9 (44%)	None
Yadav et al. [24]	^225^Ac-DOTATATE	3 (2–9)	100 kBq/kg; 8-week interval	42.4 MBq (15.5–86.6)	RECIST 1.1	22.5 months (8–30)	7/8 (87.5%)	4/8 (50%)	None
Ballal et al. [25]	^225^Ac-DOTATATE	3 (1–5)	100 kBq/kg; 8-week interval	22.5 MBq (7.8–44.4)	RECIST 1.1	8 months (2–13)	24/24 (100%)	15/24 (62.5%)	None

Legend: DCR = disease control rate; DRR = disease response rate; NR = not reported.

## Data Availability

The data presented in this study are available on request from the corresponding author.

## Supplementary Materials

The following supporting information can be downloaded at: https://www.mdpi.com/article/10.3390/medicina61081341/s1, Table S1: PRISMA checklist.
